# Strategic Surgical Intervention in a Rare Presentation of Hip-Spine Syndrome With Lumbosacral Malunion: A Case Report

**DOI:** 10.7759/cureus.66081

**Published:** 2024-08-03

**Authors:** Miyu Tamaki, Akihiko Hiyama, Daisuke Sakai, Masahiko Watanabe

**Affiliations:** 1 Department of Orthopaedic Surgery, Tokai University School of Medicine, Isehara, JPN; 2 Department of Orthopaedic Surgery, Surgical Science, Tokai University Hospital, Isehara, JPN

**Keywords:** spondylolytic spondylolisthesis surgery, sagittal alignment, adult spinal deformity, lumbosacral dysplasia, hip-spine syndrome

## Abstract

The hip and lumbar spine are closely related and can create similar patterns of pain and dysfunction. Furthermore, diagnosing and treating hip and spine conditions can be challenging due to the overlap of symptoms. This report describes the successful treatment of a 54-year-old male with hip-spine syndrome following multiple surgeries for spondylolytic spondylolisthesis. The patient presented with low back pain (LBP) and bilateral hip pain, with radiological findings indicating spinal deformity and hip joint synovitis. Two years after two-stage corrective surgery, including pedicle subtraction osteotomy (PSO), the hip synovitis resolved and the symptoms improved. This case emphasizes the need to consider hip-spine syndrome as a possible complication of lumbosacral spine fusion surgery and demonstrates the efficacy of two-stage corrective surgery with pedicle subtraction osteotomy in treating this condition.

## Introduction

Hip-spine syndrome was first reported by Offierski and MacNab in 1983 [1]. The hip and lumbar spine are closely related and can create similar patterns of pain and dysfunction. The hip-spine syndrome poses diagnostic and therapeutic challenges due to overlapping symptoms between hip and spine conditions. This paper describes a rare case of hip-spine syndrome in a patient with lumbosacral dysplasia following malunion after spondylolytic spondylolisthesis surgery at the L5 level.

## Case presentation

**Figure 1:**
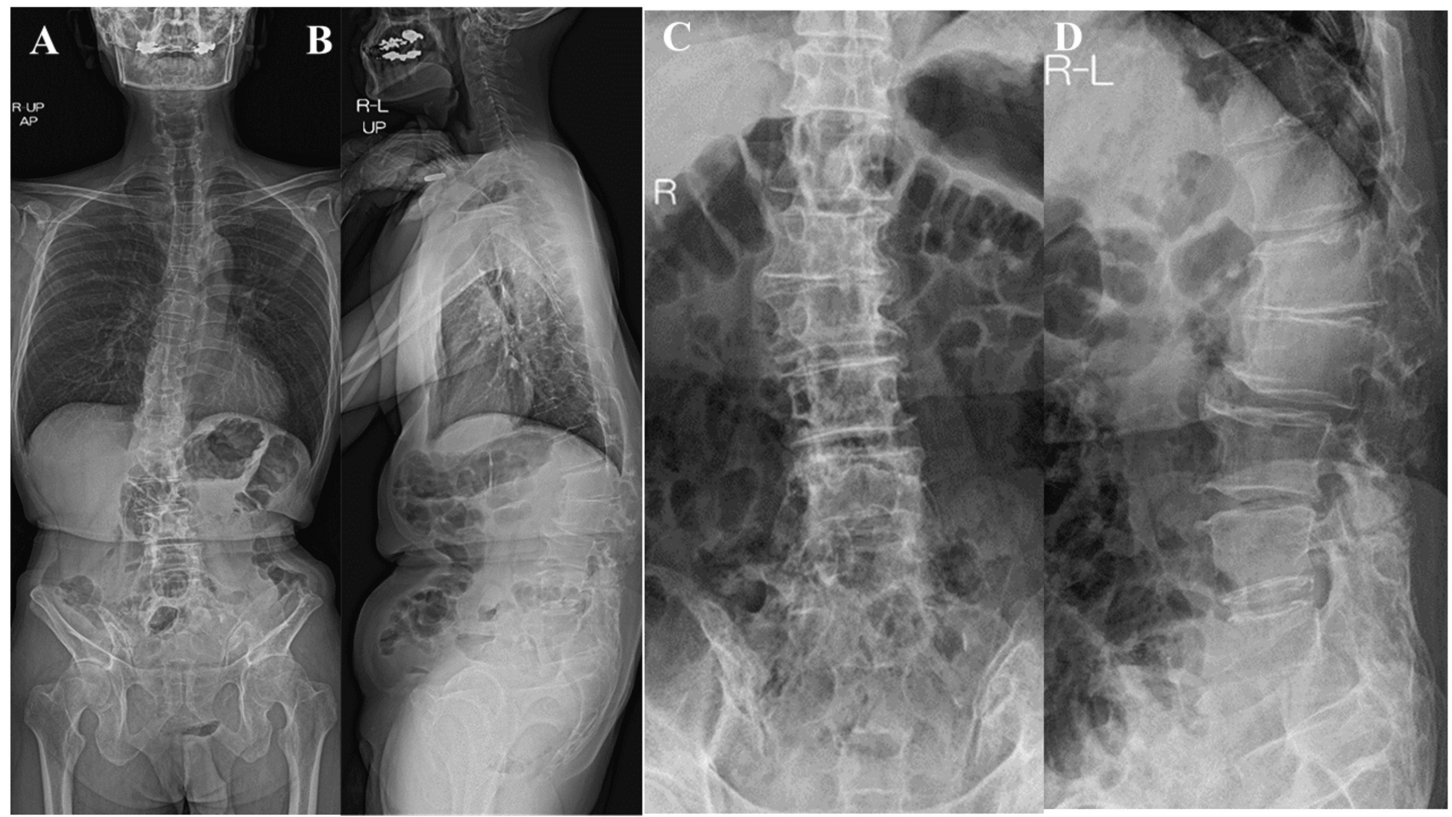
Preoperative plain X-ray imaging of the spine and hip (A) Standing anterior-posterior view of the whole spine. (B) Standing lateral view of the whole spine. (C) Preoperative plain X-ray anterior-posterior view of the lumbar spine. (D) Preoperative plain X-ray lateral view of the lumbar spine.

**Figure 2:**
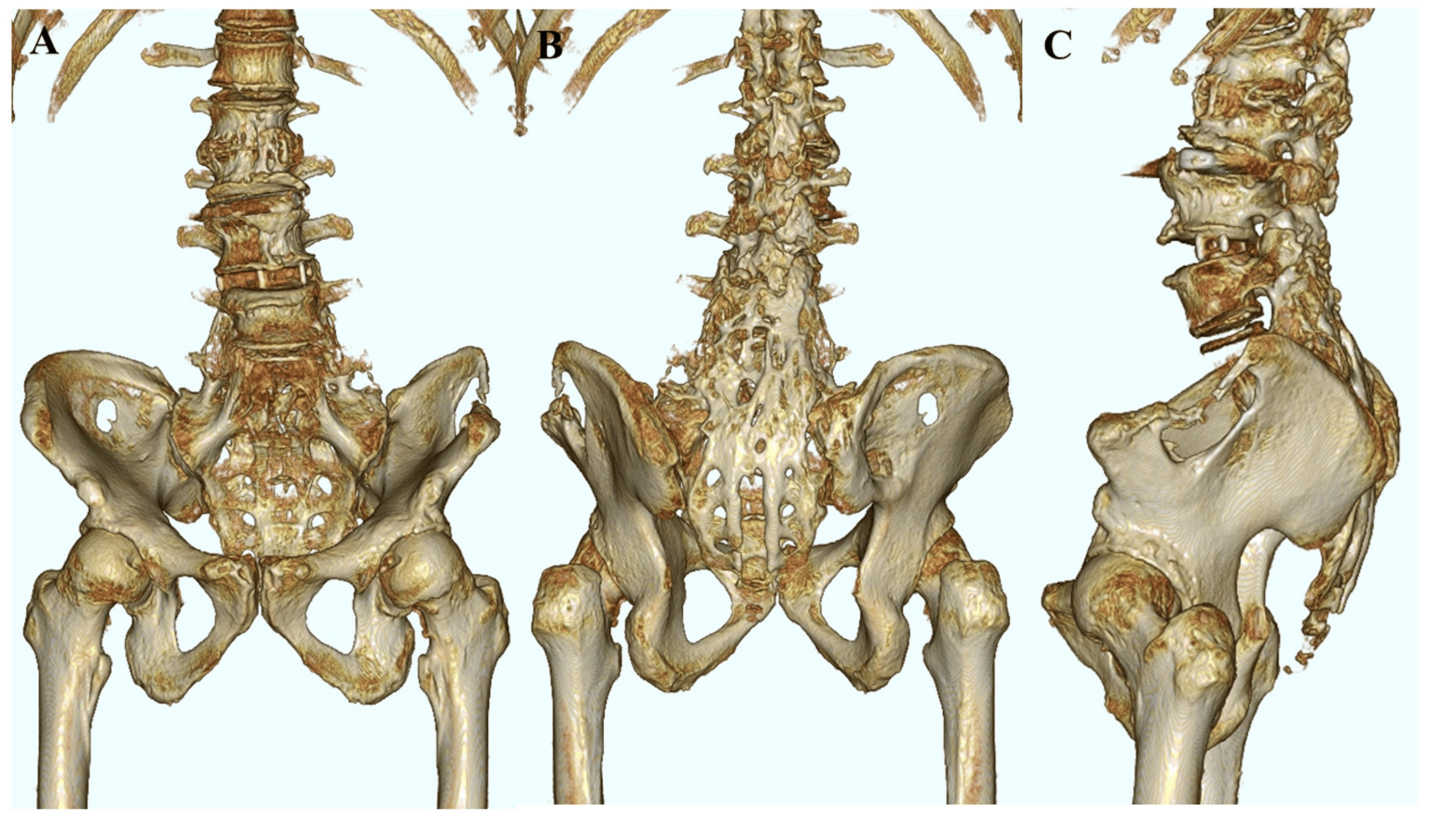
Preoperative imaging of the lower thoracic and lumbar spine (3D-CT) (A) Anterior union of the spine in the lumbar and sacral regions. (B) Posterior union of the spine in the lumbar and sacral regions. (C) Lateral union of the spine in the lumbar and sacral regions. 3D-CT: three-dimensional computed tomography

**Figure 3:**
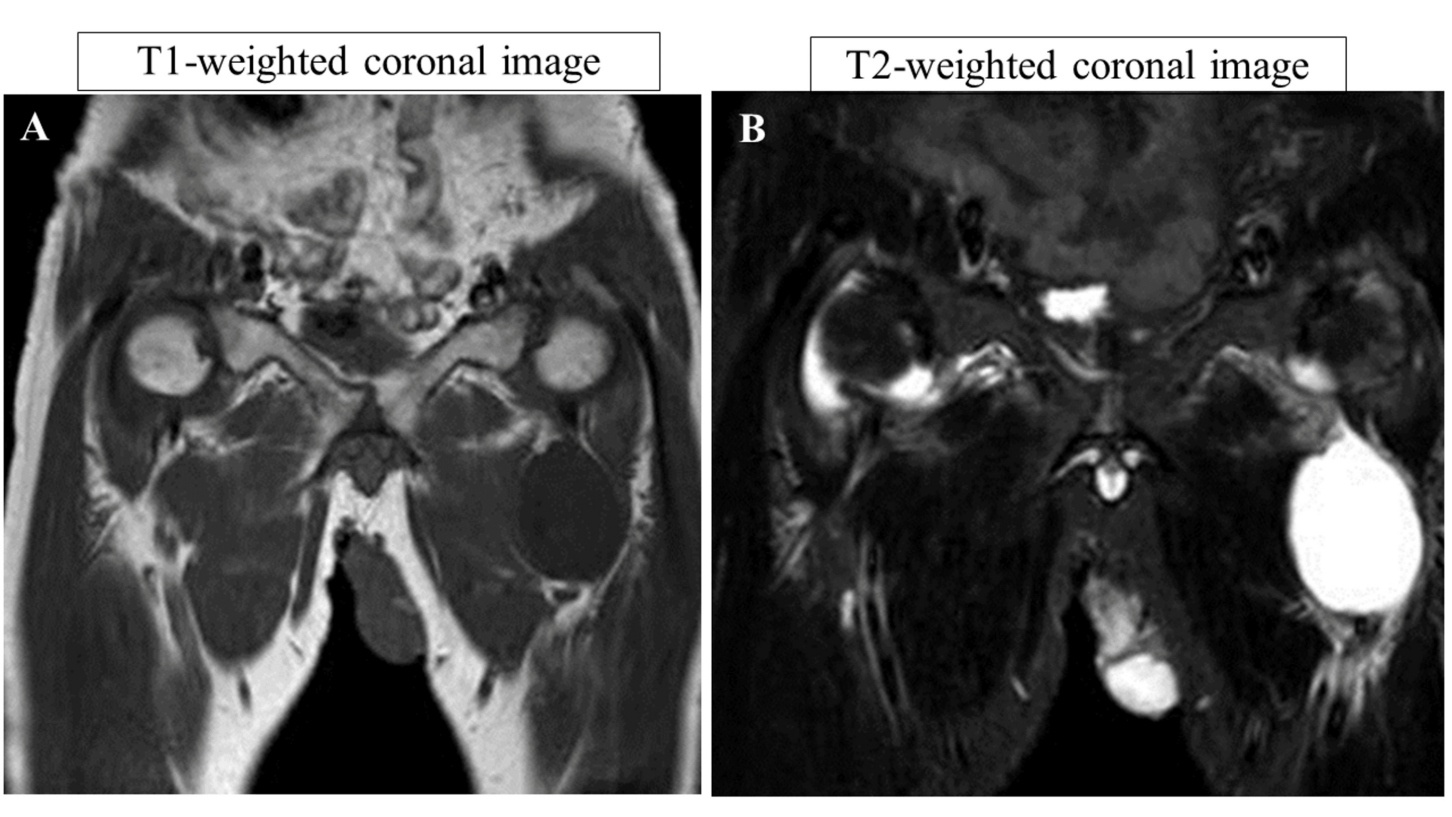
Preoperative imaging of the hip (MRI) Hip MRI scan of the hip synovial bursitis, primarily on the left side. (A) Low-intensity signal on the T1-weighted coronal image. (B) High-intensity signal on the T2-weighted coronal image. MRI: magnetic resonance imaging

A 54-year-old male presented with complaints of low back pain (LBP) and pain in both hip joints, with noted swelling in his left hip. His medical history included spondylolisthesis at the L5 level, for which he had undergone posterolateral fusion three times. Physical examination revealed pain upon flexion of both hips. X-ray images of the whole spine demonstrated bilateral degenerative hip arthritis and posterior slippage at L2 (Figure 1). Preoperative spinal parameters were measured using digitized whole-spine standing radiographs. Moreover, three-dimensional computed tomography (3D-CT) revealed lumbosacral malunion at the L5 level (Figure 2). At the same time, magnetic resonance imaging (MRI) of the hips displayed low-intensity signals on T1-weighted images and high-intensity signals on T2-weighted images, indicative of hip synovial bursitis, primarily on the left side (Figure 3).

Consequently, the patient was diagnosed with hip-spine syndrome due to a lumbopelvic alignment disorder resulting from previous surgeries, with the surgical aim being to improve femoral head coverage and correct pelvic retroversion. Considering the surgical invasiveness, corrective surgery was performed in two stages. The first stage involved a lateral lumbar interbody fusion (LLIF) at the L2/L3 and L3/L4 levels using an LLIF cage. One week later, a posterior spinal fusion with pedicle subtraction osteotomy (PSO), extending from L2 to the ilium, was performed [2]. We utilize intraoperative CT navigation for the insertion of implants, including pedicle screws. Intraoperative CT navigation facilitates the placement of bilateral pedicle screws from L2 to the ilium, and a Scoliosis Research Society (SRS)-Schwab Grade 4 osteotomy was performed at L5 [3]. This patient used a banana cage set at a 14-degree angle to achieve significant lordosis at L4/L5.

Table 1 presents the spinal parameters. Table 2 presents the Japanese Orthopedic Association Back Pain Evaluation Questionnaire (JOABPEQ) scores [4]. Table 3 presents Visual Analog Scale (VAS) scores preoperatively, six months postoperatively, one year postoperatively, and two years postoperatively. Two years postoperatively, the spinal parameters showed a notable decrease in the sagittal vertical axis (SVA) from 95.5 mm to 21.5 mm and improved lumbar lordosis (LL) from 3.6° to 20.5°. However, the pelvic incidence (PI)-LL was 46.8°, and PI-LL < 10° was not achieved. JOABPEQ scores indicated significant relief in low back pain from 0 points to 71 points and improved walking ability from 0 points to 86 points, although lumbar function decreased from 50 points to 17 points. VAS scores revealed substantial reductions in low back and leg pain, indicating successful pain management and recovery from surgery.

**Table 1:** Pre- and postoperative spinal parameters SVA: sagittal vertical axis, TPA: T1 pelvic angle, LL: lumbar lordosis, SS: sacral slope, PT: pelvic tilt, PI: pelvic incidence, TK: thoracic kyphosis

	Preoperative	Postoperative 6 months	Postoperative 1 year	Postoperative 2 years
SVA (mm)	95.5	10.9	19.7	21.5
TPA (°)	51.6	34.7	34.4	38.5
LL (°)	3.6	17.1	29.5	20.5
SS (°)	9.7	22.5	25.5	17.8
PT (°)	57.7	44.8	41.8	49.5
PI (°)	67.4	67.3	67.3	67.3
TK (°)	5.6	7.2	7.6	8.4
PI-LL (°)	63.8	50.2	37.8	46.8

**Table 2:** Pre- and postoperative JOABPEQ scores The patient is required to respond to 25 questions, from which five independent functional scores are calculated according to a predefined formula. Each functional score ranges from 0 to 100, with higher scores indicating better functional status. JOABPEQ: Japanese Orthopedic Association Back Pain Evaluation Questionnaire

	Preoperative	Postoperative 6 months	Postoperative 1 year	Postoperative 2 years
Low back pain	0	57	71	71
Lumbar function	50	25	42	17
Walking ability	0	86	64	86
Social life function	24	57	51	57
Mental health	29	54	49	54

**Table 3:** Pre- and postoperative VAS Scale ranges from 0 to 100, with lower scores indicating better functional status. VAS: Visual Analog Scale

	Preoperative	Postoperative 6 months	Postoperative 1 year	Postoperative 2 years
Low back pain (mm)	30.7	20.8	9.8	3.6
Leg pain (mm)	92.3	14.6	5.8	5.4
Leg numbness (mm)	15.3	23	3.9	0

**Figure 4:**
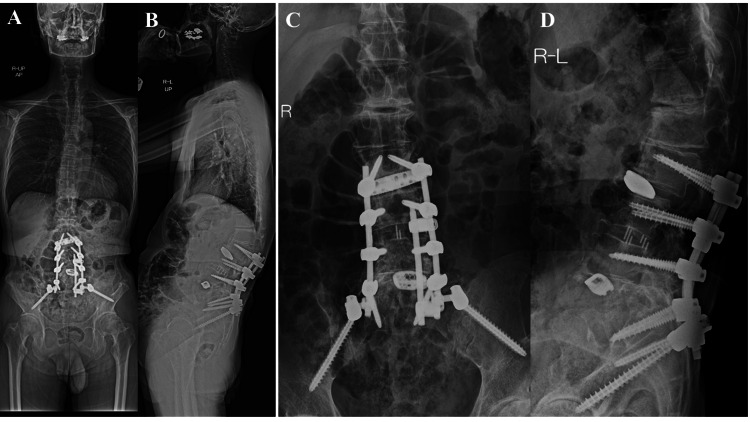
Postoperative plain X-ray imaging of the spine and hip at two years (A) Postoperative plain X-ray of the whole spine (anterior-posterior view). (B) Postoperative plain X-ray of the whole spine (lateral view) shows improved lumbosacral alignment. (C) Postoperative plain X-ray of the lumbar spine (anterior-posterior view). (D) Postoperative plain X-ray of the lumbar spine (lateral view) displays LLIF at the L2/L3 and L3/L4 levels using an LLIF cage, and posterior spinal fusion with PSO. LLIF: lateral lumbar interbody fusion, PSO: pedicle subtraction osteotomy

**Figure 5:**
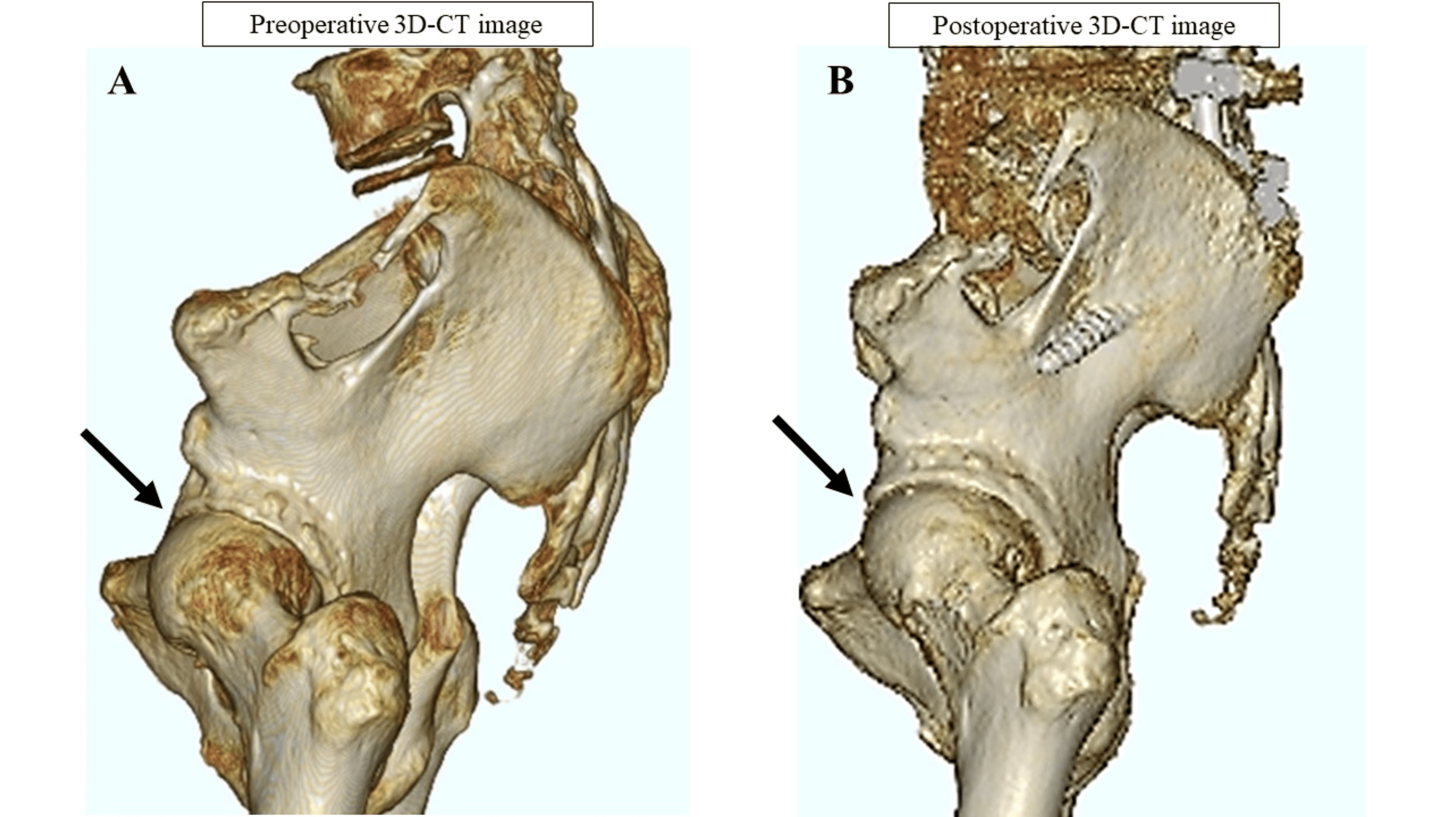
Postoperative hip imaging (3D-CT) Comparison of pre- and postoperative 3D-CT images of the lumbosacral region: The scans indicate improvements in pelvic retroversion and enhanced femoral head coverage (arrow). (A) Preoperative 3D-CT images of the lumbosacral region. (B) Postoperative 3D-CT images of the lumbosacral region. 3D-CT: three-dimensional computed tomography

**Figure 6:**
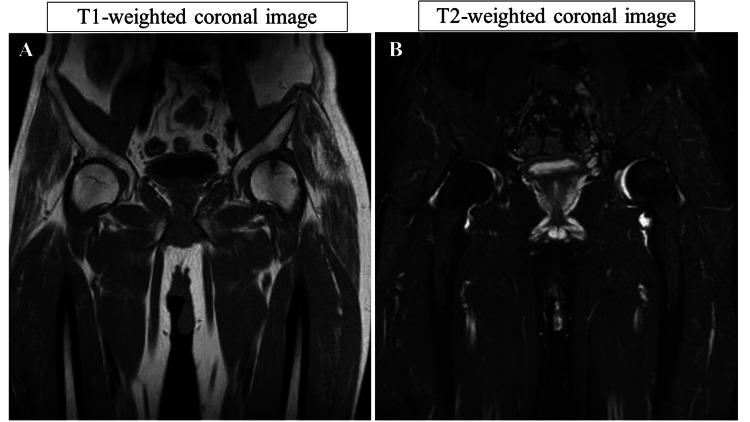
Postoperative hip imaging (MRI) (A) Postoperative hip MRI (T1-weighted coronal image). (B) Postoperative hip MRI (STIR image). Postoperative hip imaging (MRI) reveals the resolution of synovial bursitis. MRI: magnetic resonance imaging, STIR: short tau inversion recovery

The X-ray of the left hip joint two years after surgery showed no progression of hip deformity (Figure 4). Additionally, a 3D-CT scan revealed improved coverage of the acetabulum (Figure 5), and postoperative MRI indicated resolution of hip synovial bursitis (Figure 6). Follow-up assessments showed improvements in hip pain and LBP, as noted in the JOABPEQ and VAS scores.

## Discussion

Offierski and MacNab originally defined hip-spine syndrome in 1983 and classified it into four distinct types [5]. In this case, we identified lumbosacral malunion as the underlying cause of hip pain and synovitis, categorizing this case as secondary hip-spine syndrome. Multiple lumbar-sacral fusion surgeries performed for spondylolisthesis at the L5 spine fused the lumbosacral vertebrae, affecting the alignment of the spine and pelvis over time. This led to lumbar lordosis and posterior pelvic tilt (PT) changes [6]. Previous reports have indicated that an increase in PT can reduce the anterior coverage of the femoral head within the acetabulum, potentially contributing to the development or exacerbation of hip disorders [7,8]. In our case, we also observed an increased preoperative PT. As a result, there was a decrease in the anterior coverage of the femoral head, indicating an impact on the hip joint due to stress. Therefore, the surgical goal was not to correct the PILL but to address and correct the posterior PT to improve the coverage of the femoral head. Postoperatively, improvements in posterior PT were observed, enhancing the anterior coverage of the femoral head and suggesting a reduction in stress on the femoral head. This was accompanied by improvements in low back pain and leg pain.

## Conclusions

Accurate diagnosis and appropriate treatment are crucial for managing hip-spine syndrome. The present case represents a rare instance of a hip-spine syndrome caused by deformity union following multiple spinal fusion procedures performed at a young age. Corrective surgeries, including PSO at the lower lumbar spine, appear beneficial in resolving the issues associated with hip-spine syndrome related to the deformed union at the lumbosacral junction.
